# Could asymptomatic carriers spread the SARS-CoV-2 infection? Experience from the Italian second wave

**DOI:** 10.1186/s12967-021-02762-0

**Published:** 2021-03-02

**Authors:** Luigi Atripaldi, Silvia Sale, Mariaelena Capone, Vincenzo Montesarchio, Roberto Parrella, Gerardo Botti, Paolo Antonio Ascierto, Gabriele Madonna

**Affiliations:** 1 UOC Biochimica Chimica, AORN Ospedale dei Colli P.O. Monaldi, Napoli, Italy; 2 Melanoma, Cancer Immunotherapy and Development Therapeutics Unit, Istituto Nazionale Tumori IRCCS Fondazione G. Pascale, Napoli, Italy; 3 UOC. Oncologia, AORN Ospedale dei Colli P.O. Monaldi, Napoli, Italy; 4 UOC Malattie Infettive ad Indirizzo Respiratorio, AORN Ospedale Dei Colli P.O. Cotugno, Napoli, Italy; 5 Scientific Direction, Istituto Nazionale Tumori IRCCS Fondazione G. Pascale, Napoli, Italy

## Dear Editor,

We read with great interest the recent paper by Shiyi Cao et al. [1] entitled “Post-lockdown SARS-CoV-2 nucleic acid screening in nearly ten million residents of Wuhan, China” regarding the mass screening program of SARS-CoV-2 infection conducted in the metropolitan city of Wuhan, China. The authors reported the organization process, detailed technical methods used, and results of this citywide nucleic acid screening in which nearly 10 million people were recruited. This impressive screening program, conducted to evaluate the current status of infection at a given post-lockdown time point, provides a unique/extraordinary insight on the current status of the coronavirus pandemic. The authors reported 300 new asymptomatic positive cases and, in addition, 107 out 34424 previously recovered COVID-19 cases tested positive again. Interestingly, the authors highlighted that for all positive cases no “viable virus” was detected on cultures and all their close contacts tested negative for the COVID-19, which suggested the lack of evidence of transmission from asymptomatic cases. Although mass screening programs are a valid and extremely useful tools to collect important information with a high number of samples, we believe that the claims should be evaluated in the light of and compared to currently available data as well as the data cited in the article itself. There is a contradiction between the authors’ conclusions and current health recommendations for preventing the spread of COVID-19 coupled with existing literature regarding SARS-CoV-2 transmission [2]. Data suggests and has taught us [3] that even among presymptomatic patients, the high levels of viral shedding in the upper respiratory tract are a key factor in the transmissibility of the infection. In addition, the viral loads of SARS-CoV-2 were found similarly high in the four canonical symptomatic groups (presymptomatic, asymptomatic, typical symptoms and atypical symptoms); SARS-CoV-2 spreads in high concentrations from the nasal cavities prior to symptom development and viable virus was found in culture also in presymptomatic cases [4]. Additional evidence cautions on the existence of transmissibility of the asymptomatic cases as an alarm signal: He et al. argue that the transmissibility of asymptomatic cases could be lower than that of the symptomatic case [5] and Chen et al. reported no statistical difference in the transmissibility of asymptomatic cases versus symptomatic cases among close contacts [6].

**Fig. 1:**
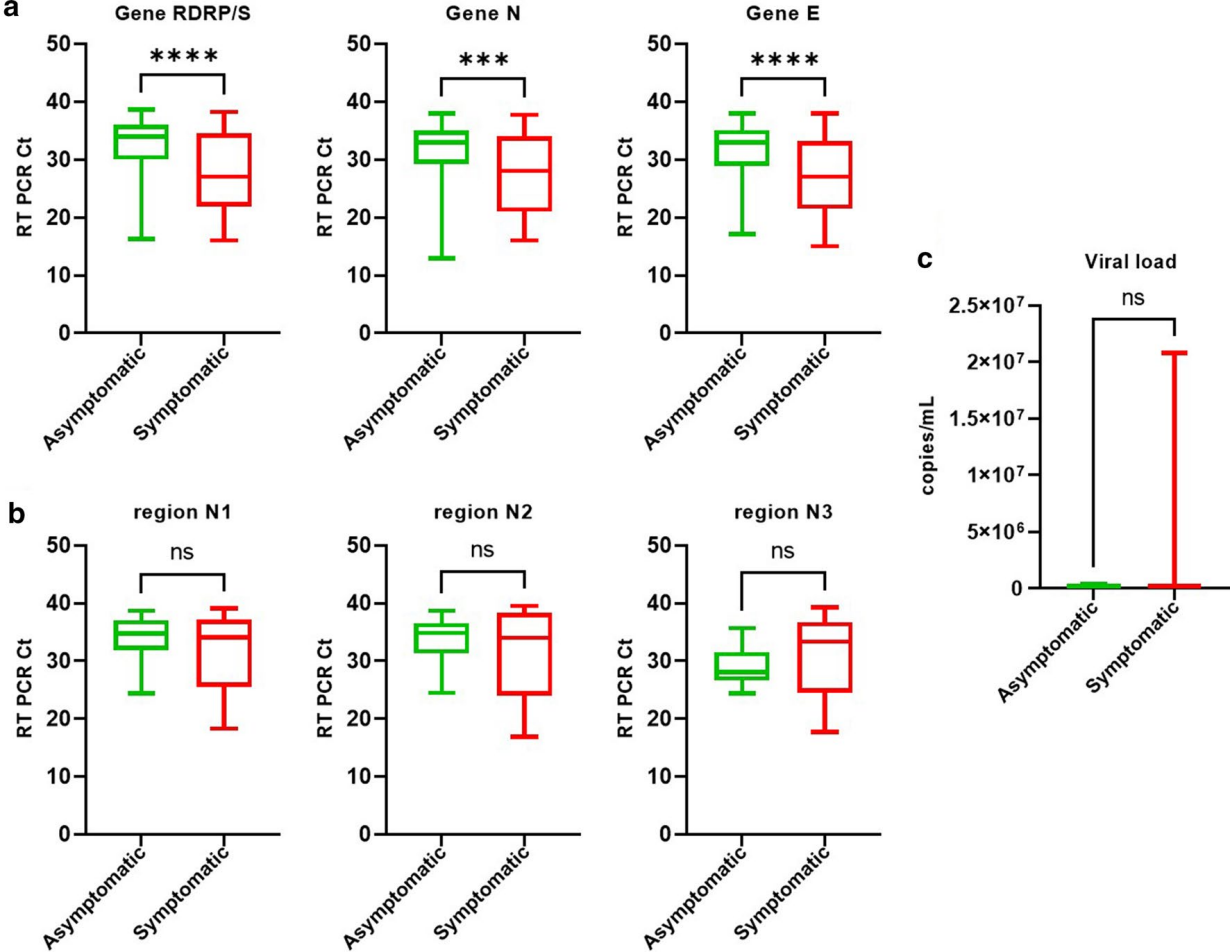
Box and Whiskers Plot of distribution of Ct data and viral load. In panel (**a**) distribution of Ct data compared to RdRP/S, N and E genes in 80 asymptomatic subjects (45 males, 35 females, median age 38.3, range 6–91) and 50 symptomatic subjects (24 males, 26 females, median age 46.7, range 6–96). In panel (**b**) distribution of Ct data compared to N1, N2, and N3 genetic regions and in panel (**c**) Box and Whiskers plot of viral load of 13 asymptomatic subjects (7 males, 6 females, median age 46.9, range 21–80) and 11 symptomatic subjects

In addition, data from our clinical experience underlines the potential involvement of asymptomatic cases in spreading Covid-19. At our Institution, ‘Azienda Ospedaliera dei Colli Monaldi-Cotugno Hospital’, Italy, 130 subjects (69males, 61females, medianage41, range6–96) were diagnosed with SARS-COV-2 infection in post-lockdown, between August 2020 and September 2020. As indicated by WHO guidelines, confirmation of SARS-CoV-2 infection was obtained by a positive RT-PCR oropharyngeal swab. We compared real-time PCR threshold cycle (Ct) data of asymptomatic and symptomatic subjects and, importantly, for a small cohort, we quantified SARS-CoV-2 RNA. We used the Allplex™ SARS-CoV-2 multiplex real-time PCR assay (Seegene Inc.) to detect 4 target genes: RdRP, S and N genes specific for SARS-CoV-2, and E gene expressed in all Sarbecovirus’ including SARS-CoV-2. A positive result (i.e., a Ct less than 40) for all viral targets indicated the presence of SARS-CoV-2 RNA in the sample. Viral load (copies/mL) detection was obtained using the Quanty COVID-19 assay (Clonit), that allows quantification of the N regions using a positive control, negative control, and known standards. A positive result (i.e., a Ct less than 40) for all three viral targets (N1, N2, and N3 genetic regions) indicates the presence of SARS-CoV-2 RNA in the sample. The quantitative detection of SARS CoV-2 RNA is left to a software which builds a standard curve with the Ct values obtained following amplification of five standards (which contain 101, 102, 103, 104, and 105 copies/μl of synthetic viral N1-encoding RNA, respectively). The viral load in the sample is calculated by interpolation of the corresponding Ct value with the standard curve. We observed a statistically significant lower real-time PCR Ct median value for the RdRP, S, N and E genes in symptomatic cases versus asymptomatic cases. Interestingly, a number of samples from asymptomatic cases showed a low real-time PCR Ct and symptomatic cases a high real-time PCR Ct (Fig. 1a, Table 1). Nevertheless, a complete overlap of the confidence intervals between the two groups was observed (Table 1). No statistically significant differences were observed between symptomatic and asymptomatic cases for real-time PCR Ct median values of the N1, N2 and N3 regions (Fig. 1b, Table 2). In addition, no notable differences in SARS-CoV-2 viral load between asymptomatic and symptomatic cases were observed. Important to note that also asymptomatic cases had marked SARS-CoV-2 viral load (Fig. 1c, Table 2). In our opinion, these data underline that also the viral loads of asymptomatic cases may be sufficient to hypothesize the transmission of the SARS-CoV-2 infection from asymptomatic subjects; measuring not only the presence but also the amount of virus in infected subjects could be precious information to help control the pandemic.

**Table 1:** Real time PCR Ct data of RdRP/S, N and E genes

	Asymptomatic (n = 80)	Symptomatic (n = 50)	95% CI	p-value
RdRP/S mean Ct (range)	32.11 (16.3–38.7)	27.64 (16–38.2)	–6.546 to –2.382	< 0.0001
N mean Ct (range)	31.39 (12.9–38.2)	27.71 (16–37.7)	–5.803 to –1.556	0.0008
E mean Ct (range)	31.49 (17.1–38)	27.03 (15–38)	–6.524 to –2.411	< 0.0001

Mean real time PCR Ct data related to 80 asymptomatic subjects (45 males, 35 females, median age 38.3, range 6–91) and 50 symptomatic subjects (24 males, 26 females, median age 46.7, range 6–96)

**Table 2:** Real time PCR Ct data of N1, N2, and N3 genetic regions and viral load

	Asymptomatic (n = 13)	Symptomatic (n = 11)	95% CI	p-value
Region N1 mean Ct (range)	34.13 (24.4–38.7)	31.4 (18.3–39.1)	–7.229 to 1.709	ns
Region N2 mean Ct (range)	33.8 (25.5–38.7)	31.1 (16.9–39.5)	–7.529 to 2.342	ns
Region N3 mean Ct (range)	29.12 (24.4–35.7)	30.8 (17.7–39.3)	–2.800 to 6.138	ns
Viral Load mean copies/mL (range)	3.09E + 04 (84.95E + 01–3.09E + 05)	2.88E + 06 (2.94E + 00–2.8E + 07)	–1635430 to 5522968	ns

Mean real time PCR Ct data and mean viral load related to 13 asymptomatic subjects (7 males, 6 females, median age 46.9, range 21–80) and 11 symptomatic subjects (6 males, 5 females, median age 63.9, range 22–96)

We strongly believe that the rapid spread of the second wave of Covid-19 is linked to the circulation of the asymptomatic or pre-symptomatic subjects in post-lockdown settings. It becomes therefore necessary to intensify mass screening programs and continue to test and quarantine all positive cases in order to prevent a potential SARS-CoV-2 third wave. To pursue this objective and continue the remarkable progress in the knowledge in the fields of prevention, treatment and management of Covid-19 disease, it is necessary to encourage scientific disclosure and continue to contribute to primary data on this “hot topic” in which still many questions remain unanswered.

## Data Availability

The datasets used and/or analyzed during the current study are available from the corresponding author on reasonable request.
